# Quantitative analysis of calcium oxalate monohydrate and dihydrate for elucidating the formation mechanism of calcium oxalate kidney stones

**DOI:** 10.1371/journal.pone.0282743

**Published:** 2023-03-09

**Authors:** Mihoko Maruyama, Koichi P. Sawada, Yutaro Tanaka, Atsushi Okada, Koichi Momma, Masanori Nakamura, Ryota Mori, Yoshihiro Furukawa, Yuki Sugiura, Rie Tajiri, Kazumi Taguchi, Shuzo Hamamoto, Ryosuke Ando, Katsuo Tsukamoto, Kazufumi Takano, Masayuki Imanishi, Masashi Yoshimura, Takahiro Yasui, Yusuke Mori

**Affiliations:** 1 Graduate School of Engineering, Osaka University, Suita, Japan; 2 Graduate School of Life and Environmental Sciences, Kyoto Prefectural University, Kyoto, Kyoto, Japan; 3 Department of Nephro-urology, Nagoya City University, Graduate School of Medical Sciences, Nagoya, Japan; 4 National Museum of Nature and Science, Tsukuba, Japan; 5 Nagoya Institute of Technology, Nagoya, Aichi, Japan; 6 Department of Earth Science, Tohoku University, Sendai, Japan; 7 Health and Medical Research Institute, National Institute of Advanced Industrial Science and Technology (AIST), Takamatsu, Kagawa, Japan; 8 Tajiri Thin Section Laboratory, Higashiosaka, Osaka, Japan; 9 Institute of Laser Engineering, Osaka University, Suita City, Osaka, Japan; Saveetha Institute of Medical and Technical Sciences: Saveetha University, INDIA

## Abstract

We sought to identify and quantitatively analyze calcium oxalate (CaOx) kidney stones on the order of micrometers, with a focus on the quantitative identification of calcium oxalate monohydrate (COM) and dihydrate (COD). We performed Fourier transform infrared (FTIR) spectroscopy, powder X-ray diffraction (PXRD), and microfocus X-ray computed tomography measurements (microfocus X-ray CT) and compared their results. An extended analysis of the FTIR spectrum focusing on the 780 cm^−1^ peak made it possible to achieve a reliable analysis of the COM/COD ratio. We succeeded in the quantitative analysis of COM/COD in 50-μm^2^ areas by applying microscopic FTIR for thin sections of kidney stones, and by applying microfocus X-ray CT system for bulk samples. The analysis results based on the PXRD measurements with micro-sampling, the microscopic FTIR analysis of thin sections, and the microfocus X-ray CT system observation of a bulk kidney stone sample showed roughly consistent results, indicating that all three methods can be used complementarily. This quantitative analysis method evaluates the detailed CaOx composition on the preserved stone surface and provides information on the stone formation processes. This information clarifies where and which crystal phase nucleates, how the crystals grow, and how the transition from the metastable phase to the stable phase proceeds. The phase transition affects the growth rate and hardness of kidney stones and thus provides crucial clues to the kidney stone formation process.

## Introduction

Kidney stones contain ~90% mineral compounds and 10% organic compounds. The mineral compounds consist of calcium stones such as calcium oxalate (CaOx) and calcium phosphate, and non-calcium stones (e.g., struvite, uric acid, cystine, proteins, and drug stones) [1]. Approximately 1.7%–14.8% of the population is affected by kidney stones once in their lifetime, and the recurrence rate at 5 years is 40% [2, 3]. Although the management of patients with symptomatic kidney stones has evolved from open surgical lithotomy to minimally invasive approaches such as percutaneous nephrolithotomy (PCNL), ureteroscopic lithotripsy (URSL), and shock wave lithotripsy (SWL), these approaches are still invasive [4]. Considering the high occurrence and recurrence rates of kidney stones, it is essential to establish the pathogenesis of kidney stone formation and devise new endourological approaches that reduce the severe rate of kidney stone disease [1, 5].

Many research groups have attempted to identify the crystal phases and proteins in kidney stones in order to clarify the pathogenesis of the disease. In most of the prior studies, the kidney stones under examination were crushed and powdered. Although the crystal phase and organic substances contained in kidney stones can be identified with the use of Fourier transform infrared (FTIR) spectroscopy, Raman spectroscopy, energy dispersive X-ray spectrometry (SEM-EDX), mass spectroscopy, and other methods [6, 7], the identification using powdering samples has had limitations; for example, the spatial information of stones, i.e., the historical data of the stones, is lost [8]. An effective way to overcome this problem is to identify the crystal phases and crystal texture classifications by using slice sections and polished thin sections of kidney stones.

Observations of such thin sections of kidney stones by optical microscopy has been conducted using slice sections and polished thin sections of kidney stones for over 70 years [9]. However, there have been problems and limitations in the technology for shaping stones, the composition analysis technology for minute regions, and the complementary analysis technology for organic and inorganic substances; the mechanisms underlying the formation of kidney stones have thus not been sufficiently clarified.

For example, deterioration of the surface structure and protein expressions occurs during the process of shaping kidney-stone thin sections. Observations and analyses of kidney-stone thin sections have become a research focus again recently, and our group developed a new method for making thin sections without severe deterioration of the surface structure or protein expressions. By using such thin sections and advanced multiple immunofluorescence staining (multi-IF staining), we were able to determine the micro-scale distributions of three different proteins [10]. The spatial distributions of these proteins in kidney stones are essential for evaluating the *in vivo* effects of proteins on kidney stone formation.

Knowledge of the precise details of the mineral distribution in kidney stones is necessary to elucidate the pathogenesis of kidney stones, including the process of stone formation process. High-resolution FTIR, Raman spectroscopy, etc., were recently introduced to observe thin sections. Castiglione *et al*. examined chemical imaging of kidney stones using a confocal Raman microspectrophotometer, and they succeeded in identifying kidney stone components, including crystal polymorphisms of CaOx and calcium phosphates [11]. Their method is helpful for determining the organization of components within stones, but it has several limitations; identifying a kidney stone containing a high concentration of proteins is difficult because of kidney stones’ intense autofluorescence background, and the quantification of each component remains a challenge [11].

The quantification of CaOx phases by FTIR has been described, and a reliable method was suggested for the bulk analysis of kidney stones [12, 13]. CaOx has four pseudo-polymorphs: calcium oxalate anhydrous, calcium oxalate monohydrate (COM), calcium oxalate dihydrate (COD), and calcium oxalate trihydrate (COT) [14, 15]. COM and COD are often found in kidney stones, and COT is occasionally present [16]. In urine, COM is in a thermodynamically stable phase, and COD is in a metastable phase (it is more soluble than COM) [17]. The potassium bromide (KBr) pellet technique and attenuated total reflection (ATR) method have enabled the measurement of small amounts of powder samples, but invasive sample preparation is required; e.g., powdering a part of a kidney stone. Blanco *et al*. reported the mapping of COM, COD, and carbonate apatite (CAP) by FTIR spectroscopy using kidney-stone thin sections [18]. They focused on the specific peaks of COM and COD (1630 cm^−1^ and 1680 cm^−1^ peaks) for the quantification of COM and COD, but the peaks were affected by other components such as phosphoric acid and organic molecules, and the mapping of the crystal phases based on these two peaks may therefore lead to incorrect results.

Yoshida *et al*. introduced a method for a more reliable phase identification of COM and COD. The method focuses on the 780 cm^−1^ peak, which is relatively unaffected by other crystal components, and Yoshida *et al*. calculated the infrared (IR) absorption of COM and COD [19]. However, this analysis method was developed for powder sample measurement and has not been expanded for microscopic FTIR measurement.

In the present study, we sought to identify and quantitatively analyze CaOx kidney stones on the order of micrometers. We first targeted the most common calcium oxalate-based stones and then performed a regional quantitative analysis of COM and COD. Kidney stones were collected from patients at Nagoya City University, and several thin sections and polished bulk samples were prepared. Polarized-light microscopy and FTIR were used for the crystal structure analysis and phase identification of the local points. The FTIR spectra were analyzed as described [19]. In addition, powder X-ray diffraction (PXRD) measurements of a small amount of powder samples collected from the thin sections were conducted. We compared the PXRD and FTIR results to establish a reliable measurement procedure, and we also analyzed a sliced bulk sample of a kidney stone by using a microfocus X-ray CT system to quantify COM and COD. The quantification results of the kidney stone samples obtained by each of these three analysis methods were consistent. The developed quantitative measurement procedures for CaOx polymorphs enabled us to investigate kidney stones in greater detail. Clarifying the polymorphisms of CaOx *in vivo* is crucial for understanding the process of kidney stone formation, and future investigations using quantitative measurements of CaOx combined with the visualization of multiple proteins in human kidney stones [10] can be expected to further elucidate the kidney stone formation processes.

## Materials and methods

### Stone collection and preparation of stone sections

CaOx kidney stones were selected from among the collection of thousands of human kidney stone samples at the Department of Nephro-Urology, Nagoya City University, Japan. The sample number of the kidney stone was #1004. Sample #1004 was partly crushed and powdered for the bulk FTIR analysis. Thin sections were prepared using the resin-embedding method. The kidney stone was wholly embedded in CaldoFix epoxy resin (Struers, Ballerup, Denmark). We manually polished the sample on glass using an aluminum oxide (Al_2_O_3_) abrasive. After each grinding and polishing step, the sections were washed to remove abrasives.

A 2-mm-thick sample was prepared for the microfocus X-ray CT system (inspeXio SMX-100CT Plus, Shimadzu, Kyoto, Japan). The remaining piece was affixed to a glass slide using Bond-E epoxy adhesive (Konishi Co., Osaka, Japan), with the polished surface facing down. We then made a second cut parallel to the glass slide with a thickness of approx. 1 mm, and the sample was ground and polished to a thickness of 20–30 μm. Since it was necessary to obtain a smoother surface for chemical analysis such as the FTIR reflection method, we polished the sample again using a diamond slurry. We prepared two thin sections of the kidney stone in this manner [20].

### Observation by polarized-light microscopy

We observed the size, coloration, and extinction of crystals in the kidney-stone thin section with the use of a polarized-light microscope (Optiphot2-Pol, Nikon, Tokyo), switching between the open-Nicol and cross-Nicol modes.

### Measurement by FTIR microscopy

We analyzed sample #1004 thin sections by using an FTIR spectrophotometer (FT/IR-6100, JASCO, Tokyo). The reflection method was used for measurement. The settings were as follows: cumulative number, 256; measurement range, 600–4000 cm^−1^, measurement area, 50-μm^2^. Because the obtained specular reflection spectrum was affected by the anomalous dispersion of the refractive index, we performed the Kramers-Kronig transformation after removing the absorption of H_2_O and CO_2_ in the atmosphere [21].

### Quantitative analysis of COM and COD using the FTIR spectra

**Fig 1:**
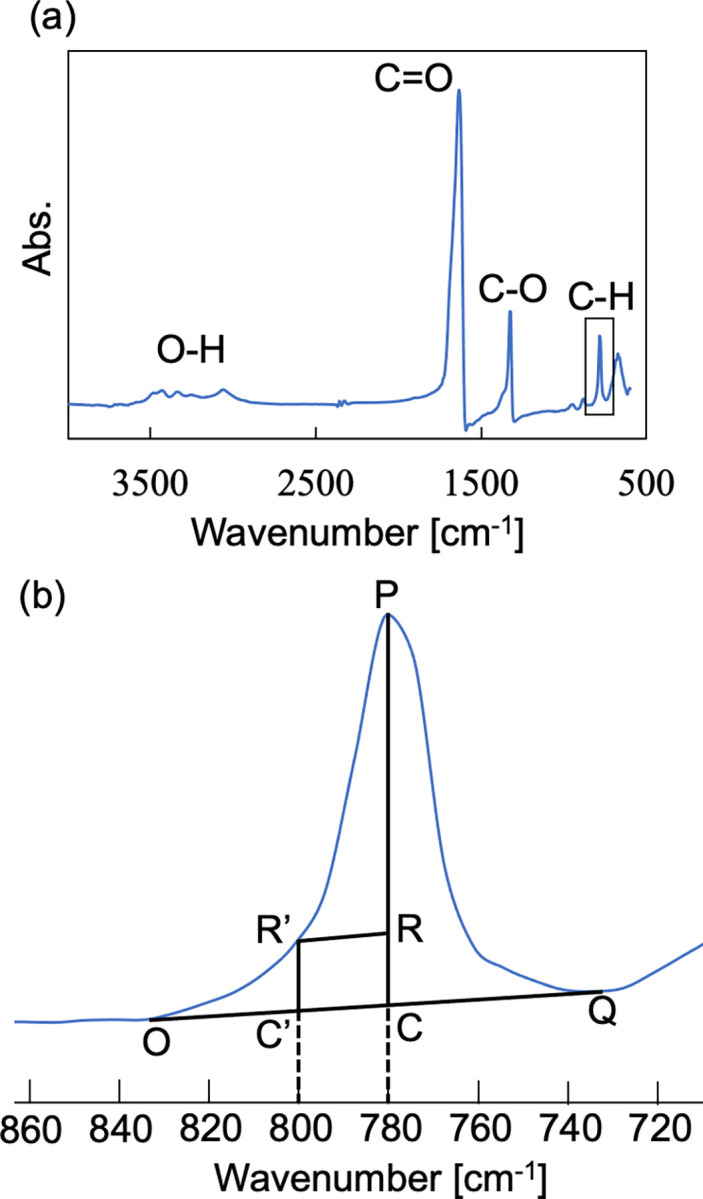
**(a)** The IR spectrum of calcium oxalate (CaOx) kidney stones. **(b)** The IR spectrum focused on a peak at 780 cm^−1^. Point P: the peak point at 780 cm^−1^. Point R’: the measurement value at 800 cm^−1^. Segment OQ: the line connecting the bottoms of both valleys of the peak. Segment R’R: the parallel line with segment OQ. Segment PR shows the amount of absorption contributed by COM, and segment RC indicates the amount of absorption contributed by COD.

The method that we applied for the quantitative analysis of powdered COM and COD using FTIR has been described [19]. In the present study, we attempted to extend this method to the analysis of kidney stones. We measured the prepared powder samples (as described in the S1 Fig) with the FT/IR-6100 spectrophotometer mentioned above. A transmission method was used for the measurement. The settings were as follows: cumulative number, 256; measurement range, 600–4000 cm^−1^, measurement area, 50-μm^2^. The absorptions of H_2_O and CO_2_ were removed from the obtained spectra to eliminate atmospheric influence. The measurement range included the O-H stretching bond at 3200–3550 cm^−1^, the C = O stretching bond around 1700 cm^−1^, the C-O stretching bond around 1300 cm^−1^, and the C-H bending bond at 675–900 cm^−1^ (Fig 1A) [22, 23].

We focused on the range of 700–900 cm^−1^. Although the infrared absorption of COM has a peak at 780 cm^−1^ and that of COD also has a peak at 780 cm^−1^, the infrared absorption of COD has a larger half-maximum width compared to that of COM. Thus, COD’s 780 cm-1 absorption peak could be fully differentiated from that of COM at approx. 800 cm^−1^. Here, we provide a summary of the analysis method in reference [19]. First, we calculated the base value of the 780 cm^−1^ peak (point C) by drawing a line (segment OQ) connecting the bottoms of both valleys of the peak (Fig 1B). We then drew a parallel line (segment R’R) with segment OQ from the measured value at 800 cm^−1^ (point R’). The vertices of lines OQ and PC are defined as point R. The ratio of line PR to line PC corresponds to the absorption contribution of COM at the 780 cm^−1^ peak, and the ratio of line RC to line PC corresponds to the absorption contribution of COD at the 780 cm^−1^ peak.

We defined the absorption ratio of COM to total calcium oxalate as *M*, and we defined the absorption ratio of COD as *D*. We describe the proportion of COM as *m* and that of COD as *d* for the calcium oxalate samples. The values of *m* and *d* can be approximated by the following linear equations, where *a*, *b*, *a’*, and *b’* depend on the apparatus used:

$$Y=\frac{M}{D}=a\times \frac{m}{d}+b=aX+b(m<d)$$

$$Y\prime =\frac{D}{M}=a^{\prime }\times \frac{d}{m}+b^{\prime }=a^{\prime }X\prime +b^{\prime }(d<m)$$

To determine *a*, *b*, *a’*, and *b’* for the FT/IR-6100 spectrophotometer, we drew a calibration curve using prepared standard samples. COM and COD were mixed at arbitrary molar ratios (see the S1 Fig). Each of the collected standard samples was measured more than six times. We measured the infrared absorption of the kidney stone samples and calculated the content ratios of COM and COD using this calibration curve.

### Measurement by powder X-ray diffraction

**Fig 2:**
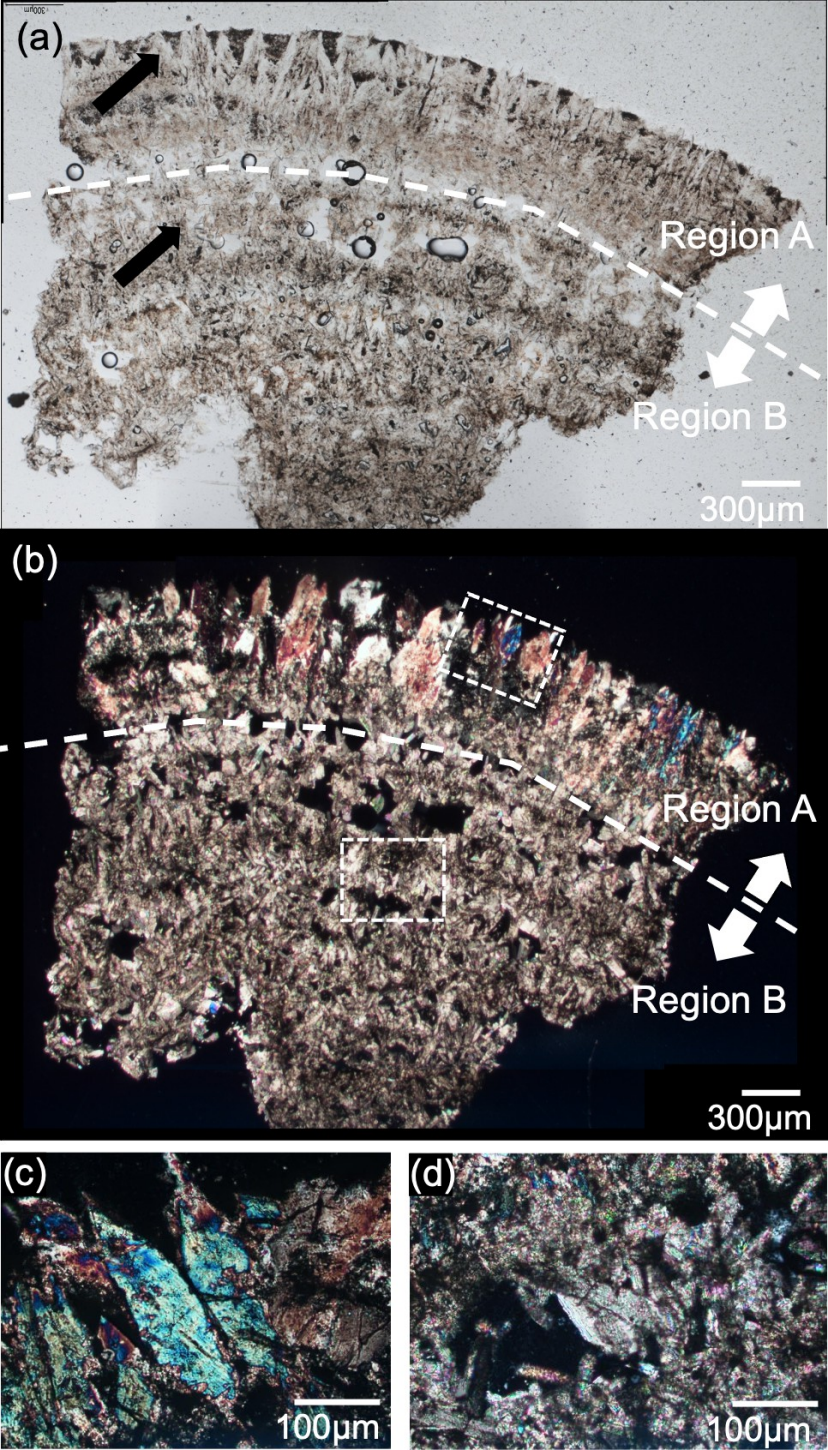
Panels **(a, b)** show an open-Nicol image and a cross-Nicol images of sample #1004 observed using a polarization microscope. Panels **(c, d)** are enlarged images of regions A and B observed by polarized-light microscopy (inside the *dashed square* in panel b).

We identified the crystal components in the kidney stones by conducting a local analysis using PXRD. After optical observation, we scraped off part of the thin section and measured the samples by PXRD. The scraped areas (approx. 50-μm^2^) are shown in Fig 2A. The PXRD data were measured at room temperature on a powder diffractometer (45 kV, 200mA, rotating anode; Rigaku SmartLab, Tokyo) in the Debye–Scherrer geometry with Cu Kα_1_ radiation monochromatized by a Johansson Ge crystal and focused by a multilayer mirror. Region B in Fig 2B was measured using Mo Kα_1_ radiation. The PXRD patterns for COM and COD were calculated using VESTA software in accord with references [24, 25].

### Microfocus X-ray CT measurements

We identified the crystal components in the kidney stones by using a microfocus X-ray CT system (inspeXio SMX-100CT Plus; Shimadzu). The measurement parameters were X-ray tube: 90 kV, 44 mA and voxel size: 0.005 mm/voxel. We measured a 2-mm-thick kidney stone sample and obtained a series of X-ray tomographic images. The main components of CaOx kidney stones, i.e., COM and COD, exhibit different amounts of X-ray absorption [26]. Approximate local phase identification was performed based on the differences in these absorption values. The amount of material can also affect X-ray absorption. Therefore, in the case of samples with crystals smaller than those that can be discriminated with an optical microscope (several micrometers or less), it is difficult to determine the ratio of COM/COD by microfocus X-ray CT system only. However, if crystal sizes are large enough to be distinguished with an optical microscope, the difference in density between COM and COD can be determined by the CT value. We thus carefully assessed the adequacy of this boundary condition by comparing it with the photographs obtained by the polarized-light microscopy. The samples used for the microfocus X-ray CT system and the samples used for the polarized-light microscopy observations were processed from the same kidney stone. The analysis procedure using the obtained X-ray tomographic images is shown in the S2 Fig.

### Ethics statement

The research project presented herein was approved by the institutional review board of the Graduate School of Medicine, Nagoya City University. All methods were carried out in accordance with the relevant guidelines and regulations. The requirement for patients’ written informed consent was waived in light of the study’s retrospective nature and use of anonymized samples.

## Results

Fig 2 provides images of a sample #1004 thin section observed by a polarization microscope; (a) is an open-Nicol image and (b) is a cross-Nicol image. The color of the crystals that make up the kidney stones ranges from light brown to brown. The color distribution was seen concentrically in the stone. The thinness of the sample (approx. 20–30 μm) and the surface smoothness made it possible to observe kidney stone samples clearly by optical methods and to analyze them by the FTIR reflection method. We divided the thin section into two areas (regions A and B), as shown in Fig 2A and 2B, because the structures and crystal sizes were different. These areas were observed and analyzed separately using various methods. The details of this process are presented below.

Panels (c) and (d) of Fig 2 show magnified cross-Nicol images of the crystals in regions A and B, respectively. Region A was composed mainly of crystals with sizes on the order of 100 μm, and the colors varied from blue to magenta with a thickness of 20–30 μm. The color tone faded as the observation stage was rotated and then disappeared entirely at an extinction angle of ~45°. With further rotation, the crystals’ brightness faded from ~45° and showed maximum intensity when the rotation angle was ~ 90°. The optical behavior indicated that large crystals (100 μm in size) were almost single crystals. Region B was composed of crystals with dimensions on the order of 10 μm, and the color was iridescent with a thickness of 20–30 μm because of internal interference. Determining the extinction angle of each crystal is difficult because of the iridescent color.

**Fig 3:**
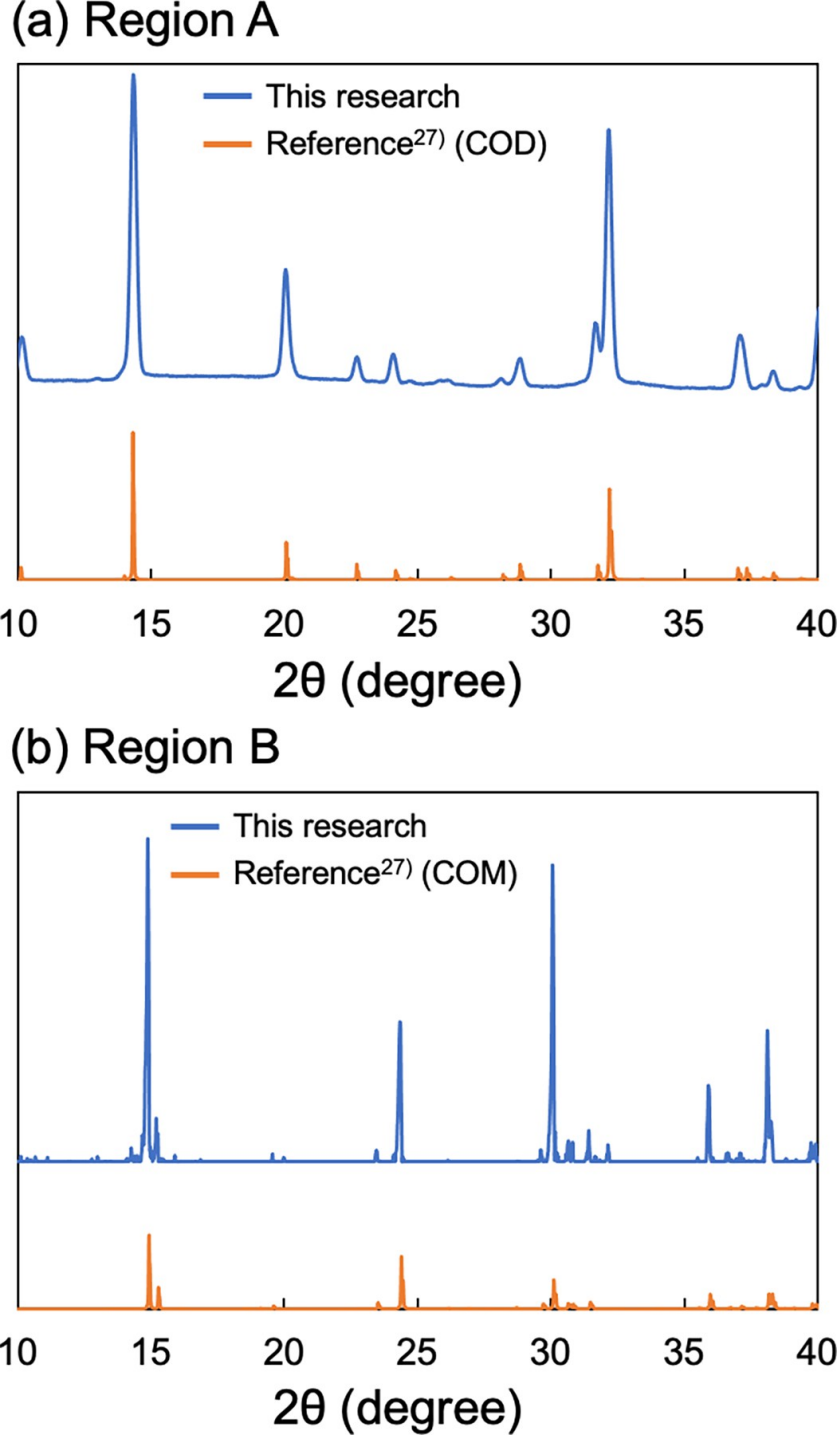
The measurement result of kidney stone sample #1004 by PXRD. **(a)** The region A results. **(b)** The region B (shown in Fig 2) results. *Upper graphs*: The COM or COD result obtained in the present study. *Lower graphs*: The COM or COD results described in reference [27].

We performed local sampling from the thin section of the same sample for the phase identification of small regions by using PXRD. Fig 3 shows the PXRD patterns of regions A and B. The PXRD pattern of region A (Fig 3A) showed that there were specific peaks of COD (peaks at ~14.3°, 20.1°, and 32.2°) and no specific peaks of COM (peaks at ~14.9°, 24.4°, and 30.1°). The PXRD pattern of region B (Fig 3B) shows specific peaks of COM; no specific peaks of COD were detected. These results confirmed that region A was composed mainly of COD crystals, and region B was composed mainly of COM crystals.

**Fig 4:**
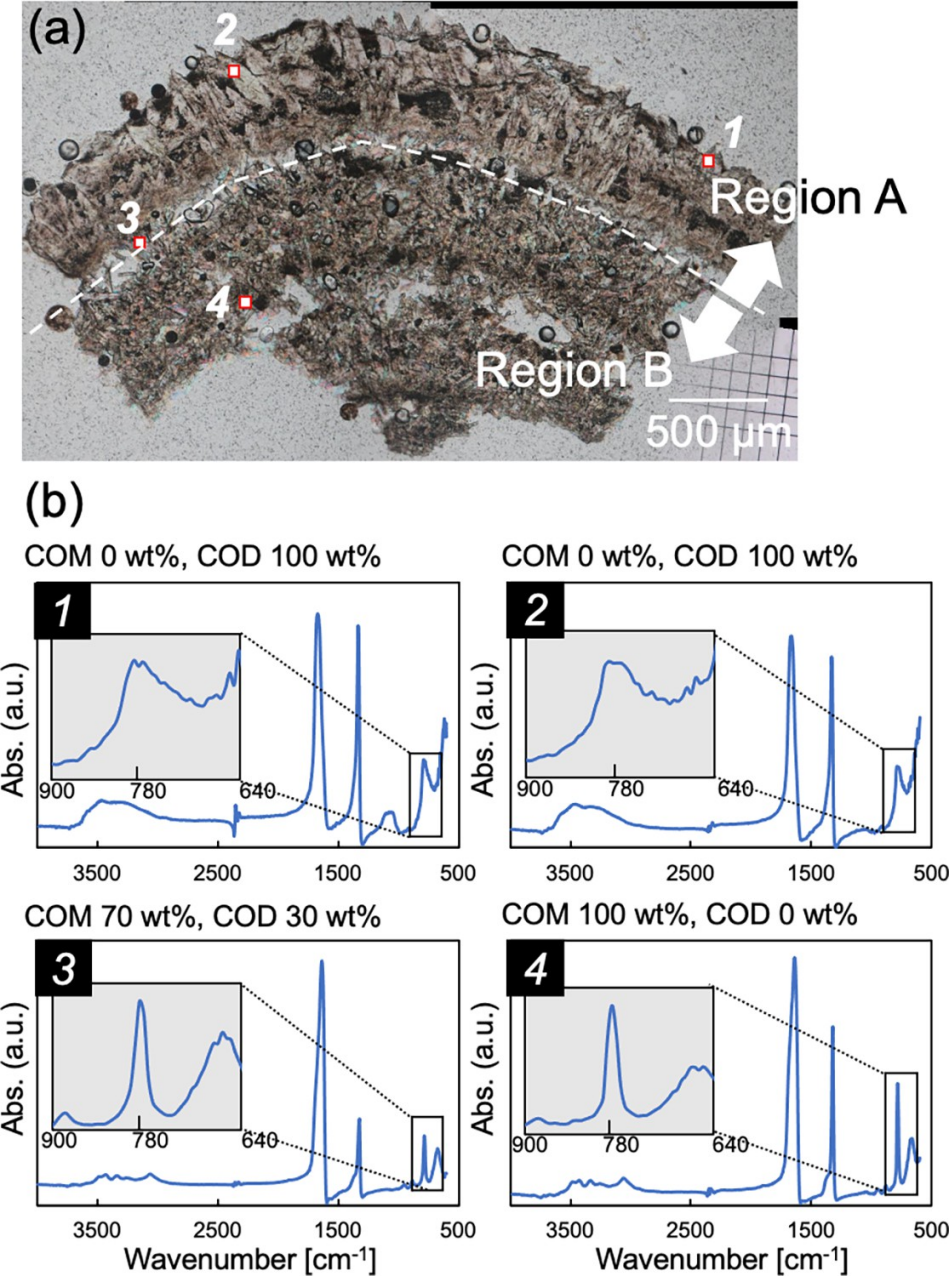
**(a)** An open-Nicol image of the second section of sample #1004. *Red marks and numbers*: the FTIR measurement points. **(b)** The measurement results for each measurement point obtained by FTIR microscopy. The *inner graphs* are the enlarged views of the peaks at 780 cm^−1^ in each graph, and the ratio of COM to COD in the upper part of the graph shows the ratio calculated by the analytical method derived in this study.

Fig 4A shows an open-Nicol image of the thin section of sample #1004. Note that the thin section was the second shaped from the same sample #1004 kidney stone. Although the shape is different from that shown in Fig 2, the second thin section was also divided into two regions (A and B) based on observations by polarized-light microscopy. The FTIR measurement points in the image are indicated by solid squares with numbers (from *1* to *4*). Fig 4B shows a set of IR spectra at each measurement point. The peaks of CaOx, as shown in Fig 1, were also identified in each spectrum. The absorption features derived from the O-H band (3200–3550 cm^−1^) and the absorption on the lower wavenumbers (the C = O stretching bond around 1700 cm^−1^, the C-O stretching bond around 1300 cm^−1^, and C-H the bending bond at 675–900 cm^−1^) indicated that the main phase at measurement points *1* and *2* were COD and the main phase at measurement points *3* and *4* were COM [23].

To obtain more details on the ratio of COM to COD, we analyzed the spectrum focusing on the peak near 780 cm^−1^ (Analysis 1). We confirmed that the COD ratio was 100±10 wt% at measurement points *1* and *2*. This established that the main CaOx phase in region A is COD. In contrast, the COM ratio at measurement point *4* was 100±10 wt%, demonstrating that the main CaOx phase in region B is COM. At the boundary between regions A and B, similar to measurement point *3*, the crystal phases observed in regions A and B were mixed. According to our calculations, the estimated ratio of COM was 70±10 wt%, and that of COD was 30±10 wt% from the IR spectrum. For comparison, we analyzed the FTIR spectra using the methods described in reference [28] (Analysis 2) and reference [12] (Analysis 3). Martin *et al*. (ref. [28]) focused on the absorption bands at 910 cm^−1^ and 780 cm^−1^. Mauric-Estepa (ref. [12]) focused on the peak shift of the absorption at 1324 cm^−1^. The results calculated using these three methods are summarized in Table 1.

**Table 1:** The ratio of COM and COD calculated by the three different analyses.

	Analysis 1 (Ref. [19])	Analysis 2 (Ref. [28])	Analysis 3 (Ref. [12])	PXRD
	(780 cm^-1^)	(910 and 780 cm^-1^)	(1324 cm^-1^)	
	COM COD	COM COD	COM COD	
①	0% 100%	50% 50%	50% 50%	COD
②	0% 100%	57% 43%	63% 47%	COD
③	70% 30%	100% 0%	48% 52%	-
④	100% 0%	100% 0%	54% 46%	COM

The results of Analysis 2 tended to indicate a lower COD amount compared to the results of Analysis 1. The results of Analysis 3 showed that the ratio of COM to COD was approximately half at each of points *1* to *4*. The PXRD results showed that region A (containing measurement points *1* and *2*) is composed mainly of COD, whereas region B (containing measurement point *4*) is composed of mainly COM. The results of Analysis 2 showed that measurement areas *1* and *2* contained 50%–57% COM, and Analysis 3 also showed 50%–63% COM, but these values were not consistent with the PXRD results. The results of Analysis 1 matched well with the PXRD dataset.

**Fig 5:**
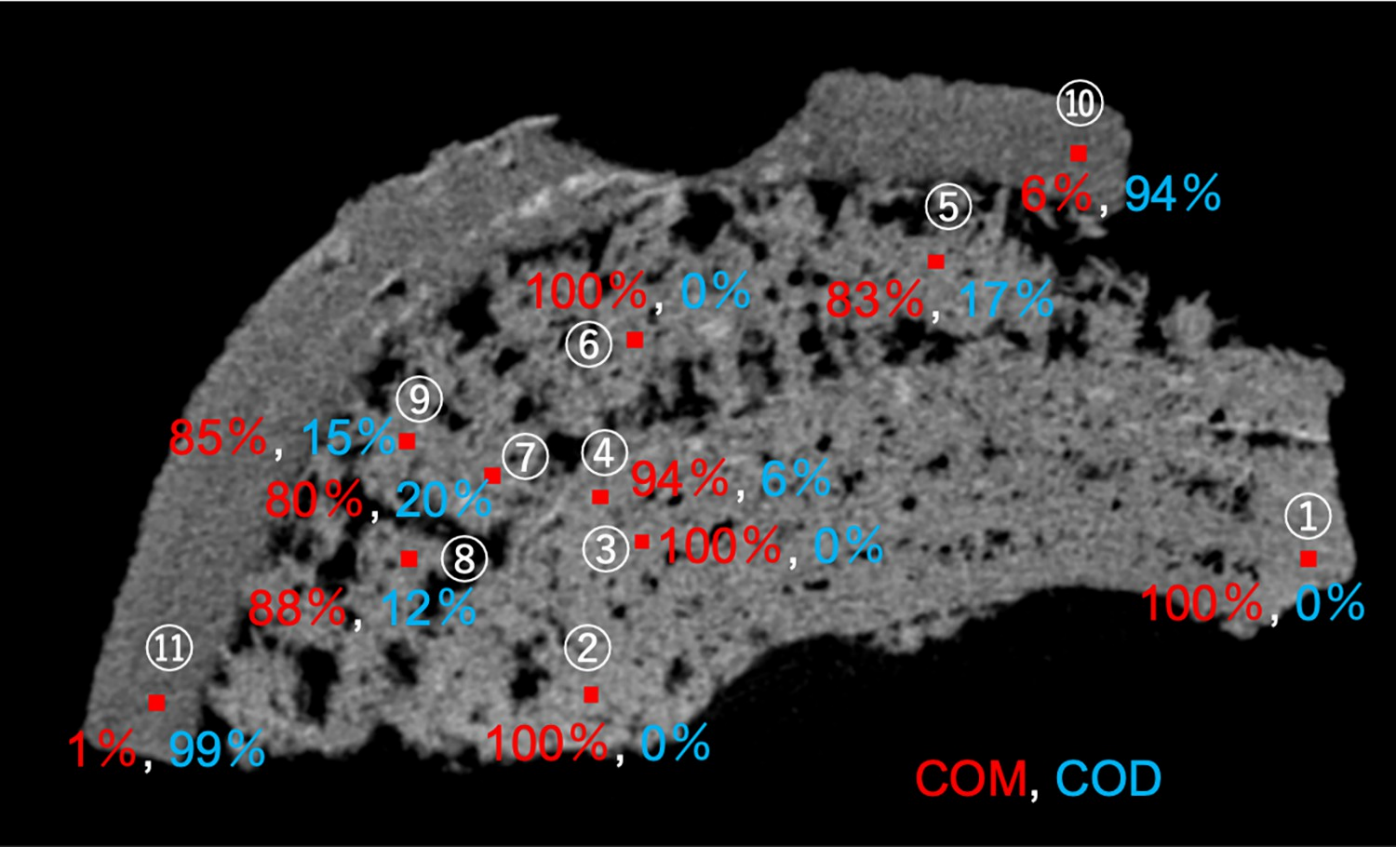
A microfocus X-ray CT image. Eleven analysis points are indicated with *red solid squares* and *numbers*. The analyzed results (the ratio of COM and COD in the 50-mm^2^ squares) are also shown. *Red numbers*: the COM ratio. *Blue numbers*: the COD ratio for each area.

Fig 5 provides a series of tomography images of sample #1004 obtained using the microfocus X-ray CT system. To obtain further details about the ratio of COM to COD, we analyzed 11 measurement points as shown in Fig 5. Each measurement point was 50-μm^2^ and is shown as a solid red square. Our calculations showed that the ratio of COD of the measurement points in Region A (10, 11) was >90%. The percentage of COD of the measurement points in Region B (1–4 and 6–8) was <10% (in other words, the ratio of COM was >90%), with the exception of points 7 and 8. The percentages of COM and COD at points 7 and 8 were 80% and 20% for point 7 and 88% and 12%, respectively for point 8. The ratios of COM and COD at points 5 and 9, which were located between region A and region B, were 83% and 17% for point 5 and 85% and 15%, respectively, for point 9. Points 7 and 8 were also located near the boundary areas of regions A and B and thus exhibited relatively high COD rates.

The observation and analysis results of the microfocus X-ray CT system were almost the same as the results obtained by PXRD and microscopic FTIR (Analysis 1). Notably, we were able to obtain a sequence of similar CT images in the measurements by the microfocus X-ray CT system. Thus, by performing the same analysis on each image of a sequence, it is also possible to calculate the total ratio of COM and COD contained in an undamaged kidney stone sample. It has been reported that the results of kidney stone composition analyses differ depending on the FTIR equipment and/or the reference spectrum [29]. It is thus essential to evaluate the validity of the reference spectrum by different methods in this field of research, and doing so can be expected to improve the analysis accuracy in future clinical practice.

## Discussion

As described above, we succeeded in the quantitative analysis of COM/COD in 50-μm^2^ areas by using FTIR microscopy for thin sections of kidney stones and by using a microfocus X-ray CT system for bulk samples of kidney stones. Roughly consistent results were obtained by (*i*) the PXRD measurements with micro-sampling, (*ii*) the FTIR microscopy analysis of a thin section, and (*iii*) the microfocus X-ray CT system observation of a bulk sample of a kidney stone, indicating that all three methods can be used in a complementary manner. By appropriately applying these methods considering the sample shape and/or sample processing, it will be possible to study kidney stones with higher accuracy than in previous studies.

We considered the formation mechanism of sample #1004 in light of our above-described results. With the cross-Nicol image shown in Fig 2B, we observed that region B had many voids. The stone’s central part (region B) was composed mainly of COM, and the outer part (region A) was comprised mostly of COD. Generally, the center part of a kidney stone is the oldest and the outer part is the newest, similar to the growth rings of trees. We had initially assumed that region B of the kidney stone was older and region A was more recent; however, the phase transformation must be considered. Sivaguru *et al*. reported that COD dissolves and transforms into COM in the human body and that when the phase transformation from COD to COM occurs, a volume change occurs due to the discharge of water [30]. If COM crystals nucleate and gather together in the early stage of stone formation, it would be difficult for COD crystals to nucleate on the surface of COM crystals because of the solubility and supersaturation difference between COM and COD.

In the human body’s internal environment, the solubility of COD is higher than that of COM; this means that the supersaturation of COM is always higher than that of COD [17]. A more acceptable hypothesis regarding the formation of kidney stones is thus as follows. At the early stage of stone formation, COD crystals nucleate and grow, and then COD gradually transforms into COM from the center part of the COD stone. The previous nucleation of COD is a natural phenomenon, because when supersaturation is sufficiently high for both the stable phase and the metastable phase, the crystals in the metastable phase can nucleate preferentially because of the lower interface energy at this phase (Ostwald’s step rule) [31]. Similar phenomena often occur in other materials [32, 33].

The transformation from COD to COM is mainly a solution-mediated transformation [30]; dissolutions of COD crystals are needed. However, supersaturations of COM and COD in urine are always high enough for CaOx crystals to grow. Where can COD dissolve in urine? The answer is: the inside of a kidney stone. The surface of a kidney stone is constantly exposed to urine, which is a highly supersaturated environment; thus, there is no chance of dissolving COD and COM. In the case of a COD kidney stone, many COD crystals gather and the stone has many gaps between crystals. The center of the stone becomes a semi-closed system. Many gaps between crystals let urine enter inside the stone where COD crystals can continue to grow using Ca^2+^, C_2_O_4_^2−^, and other solutes. The flow inside of the kidney stone is stagnated because of the narrow passages between crystals; the supply of solutes thus sometimes becomes insufficient. If there are only COD crystals, the crystal growth stops when the supersaturation of COD reaches nearly the equilibrium point.

However, in most cases, COM, which is the stable phase of CaOx in the human internal environment, nucleates following COD crystals’ nucleation and growth. If the supersaturation of COD is near equilibrium and COM crystals co-exist, COM can still grow and Ca^2+^ and C_2_O_4_^2−^ are consumed, and then finally the solution will reach an undersaturated state for COD crystals. At such an area, COD will partially dissolve, and Ca^2+^ and C_2_O_4_^2−^ will be released. The released solutes will be immediately consumed by COM near the dissolving COD. By this cycle, the transformation from COD to COM will gradually proceed. The solution-mediated transformation from a metastable phase to the stable phase often occurs in other materials [34–36].

Finally, the mosaic texture composed of irregularly oriented COM crystals appears from the center part of the kidney stone (Fig 2D). The transformation stops or significantly slows down at sites where the supply of solutes from urine and the consumption of solutes by COM growth are balanced. In our sample, the boundary between regions A and B was probably such areas (Figs 2A, 2B and 5). The supply of solutes for COD and COM is great enough outside of the boundary; we thus often observe kidney stones that have a COM core area and a COD rim area [23].

CaOx in kidney stones partially dissolves in the human body [30], but the dissolution process is followed by a phase transformation from COD (a metastable phase of CaOx) to COM (the stable phase of CaOx). Through this transformation, a kidney stone becomes more durable, and its structure is likely to become tight and rigid. This is a severe process in the pathogenesis of kidney stones, and further research is necessary to determine all of the details of the mechanisms underlying the formation of kidney stones. The final structure of kidney stones and/or the phase transformation speed estimated from the structure suggests the history of kidney stone growth and changes in the body’s urine environment. With a combination of the approaches described herein together with our recently developed analysis technique, i.e., multicolor imaging of calcium-binding proteins in human kidney stones [10], various kidney stone samples can be evaluated, and the information obtained about the stones will reveal the factors of the processes of stone formation and the interaction with organic molecules.

## Conclusion

We performed the phase identification and a quantitative analysis of CaOx kidney stones on the order of micrometers, maintaining their spatial information. Polarized-light microscopy FTIR, and PXRD measurements were performed for the crystal structure analysis and phase identification of local points using a thin section of a kidney stone. We also conducted a quantitative analysis of CaOx in a sliced bulk sample of a kidney stone by using a microfocus X-ray CT system. The extended analysis of the FTIR spectrum, focusing on the 780 cm^−1^ peak, made it possible to perform a reliable analysis of the COM/COD ratio. Using polarized-light microscopy, we mapped the two-dimensional distribution of crystal phases with a reliable COM/COD ratio. The data from the microfocus X-ray CT system also gave us a reliable quantitative COM/COD ratio using the bulk sample of a kidney stone.

Our analytical protocol minimizes damage during measurements to delicate stone samples containing various crystal phases and proteins. Since the quantitative results obtained by FTIR microscopy, microfocus X-ray CT, and PXRD are roughly consistent, each method showed slight sample damage (specifically, the phase transition of crystals and denaturation of organic substances). The results demonstrated that the quantitative reliability of each analysis was reliable. This method enables us to discuss in more detail the locations where crystal nucleation and phase transition occur, how such processes advance, and the environmental changes during the processes. The polymorphism of CaOx *in vivo* is crucial for understanding the processes of kidney stone formation. Further investigation using quantitative measurement protocols for CaOx combined with the visualization of multiple proteins in human kidney stones [10] will elucidate the kidney stone formation processes. We believe that this new methodology, examining the crystal phase and quantifying CaOx, will help reveal the time course of kidney stone formation together with environmental changes that accelerate the process.

## Supporting information

S1 Fig PXRD pattern of COD powder used as a standard sample by PXRD. (TIF)

S2 Fig A microfocus X-ray CT image and the analysis procedure. (TIF)
