# Supplementary figures and images for: Quantitative analysis of calcium oxalate monohydrate and dihydrate for elucidating the formation mechanism of calcium oxalate kidney stones
Source: PLoS One. 2023 Mar 9;18(3):e0282743. doi: 10.1371/journal.pone.0282743 (PMC9997882; doi:10.1371/journal.pone.0282743)

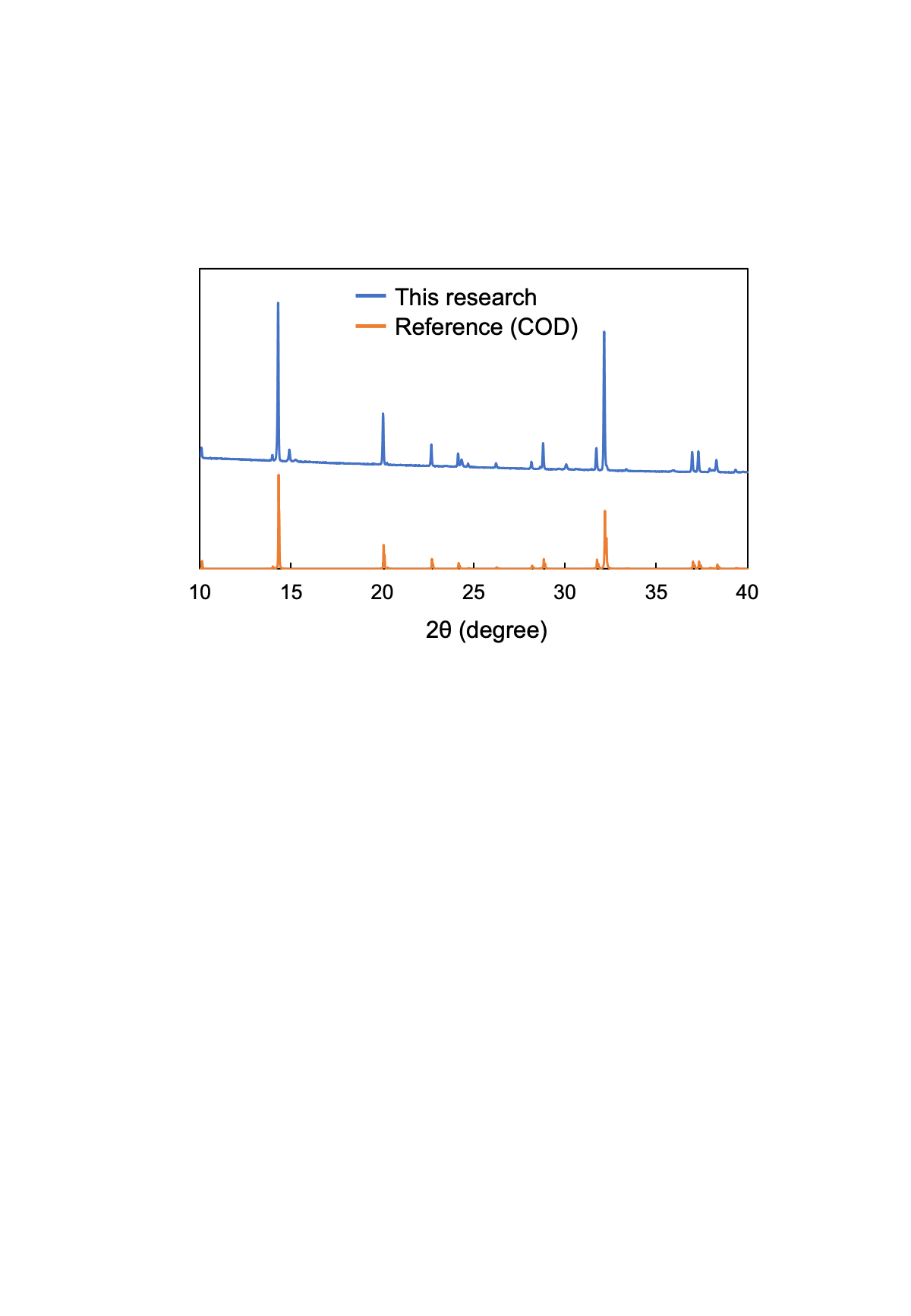

Supplement: S1 Fig — (TIF) [file pone.0282743.s001.tif]

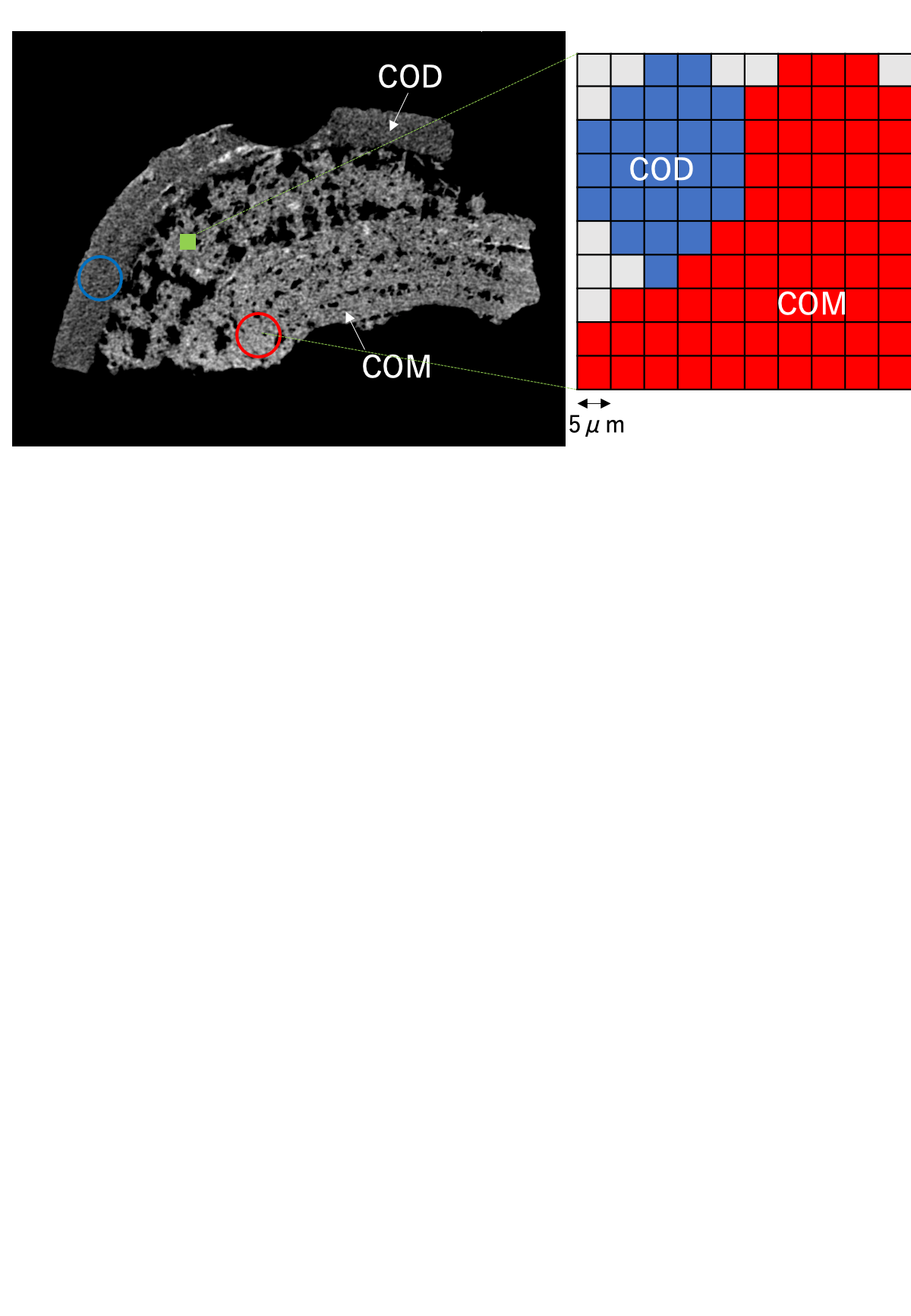

Supplement: S2 Fig — (TIF) [file pone.0282743.s002.tif]
